# Prevalence of dysmenorrhea among University students in Northern Ghana; its impact and management strategies

**DOI:** 10.1186/s12905-018-0532-1

**Published:** 2018-02-13

**Authors:** Evans Paul Kwame Ameade, Anthony Amalba, Baba Sulemana Mohammed

**Affiliations:** 1 Department of Pharmacology, School of Medicine and Health Sciences, University for Development Studies, P.O.Box TL 1350, Tamale, Ghana; 2 Department of Health Professions Education and Innovative Learning, School of Medicine and Health Sciences, University for Development Studies, Tamale, Ghana

**Keywords:** Dysmenorrhea, Prevalence, Students, Management, Impact, Ghana

## Abstract

**Background:**

The period of menstruation is an eventful one for a significant number of post-pubescent females as they experience lower abdominal pains referred to as dysmenorrhea. This study conducted among female students of the Tamale campus of the University for Development Studies assessed the prevalence of dysmenorrhea, its impact on the students and treatment methods applied.

**Methods:**

A cross-sectional study using a self-administered questionnaire was used to obtain data from 293 randomly selected female students. Data was analyzed using Graph Pad 5.01. Association between different variables was tested.

**Results:**

The prevalence rate of dysmenorrhea was 83.6% with more than half describing their pain which lasts less than 3 days as moderate. This dysmenorrhea during menstruation affects the daily activities of up to 61.2% of respondents. Lower chronological age (χ^2^ = 8.28; df = 2; *p* = 0.016) and gynecological age (χ^2^ = 10.09; df = 2; *p* = 0.006) were the factors that were significantly associated with the presence of dysmenorrhea. Chronological and gynecological ages, age at menarche, menstrual duration or flow level do not influence the severity of dysmenorrhea but irregular menstrual flow is significantly associated with severe dysmenorrhea (χ^2^ = 10.54; df = 2; *p* = 0.005). Only 16.3% ever reported their dysmenorrhea to the hospital but increasing pain level is significantly associated with respondents visiting a hospital (χ^2^ = 65.61; df = 2; *p* < 0.0001) or use an allopathic medication (χ^2^ = 32.77; df = 2; *p* < 0.0001). Paracetamol preparation was the most common medication used notwithstanding the severity of the pain.

**Conclusions:**

There is high prevalence of dysmenorrhea among the female students of the Tamale campus of the University for Development studies which negatively affects the daily activity of majority of them. Although, bed rest was the most common treatment method practised, paracetamol preparation was the most common allopathic drug used in self- management of their dysmenorrhea.

## Background

The onset of puberty in the life of a young girl sets into motion hormonal, psychological, cognitive and physical changes which transforms the girl from a child to a sexually matured woman [1]. Menstruation, which is controlled by the hormones of the hypothalamopituitary axis and is one of the milestones of puberty in girls, involves the cyclical shedding of the inner lining of the uterus [2]. Onset of menstruation is celebrated in some cultures as it shows the girl is becoming a woman [3]. It however, also heralds a period of inhumane treatment of some post pubescent girl since some cultures and religions consider the menstruating woman as impure leading to forced seclusion, reduced mobility, as well as dietary and social restrictions [2–5]. Some women, before or during menstruation also had to contend with dysmenorrhea which is a painful cramping sensation in the lower abdomen and sometimes accompanied by headache, dizziness, diarrhea, bloated feeling, nausea and vomiting, backache and leg pains [6–9]. This menstruation associated pain occurs as a result of excessive production of prostaglandins in the endometrium during the ovulatory cycle which then causes contraction of myometrium, vasoconstriction as well as sensitization of nerve ending [10, 11]. Dysmenorrhea is classified as primary when there is no evidence of pelvic or hormonal pathology but is secondary when the pain is due to identifiable pathological conditions including endometriosis, ovarian cysts, pelvic inflammatory disease, myomas or intrauterine devices [9, 12]. Whereas the management of secondary dysmenorrhea requires the treatment of the primary cause, non-pharmacological methods which include fatty diet restriction, exercise, rest, heat application, spinal manipulation, acupuncture, have been reported in several studies to ease the pains of primary dysmenorrhea [1, 12–14]. Medications which provide relief for primary and secondary dysmenorrhea include non-steroidal anti-inflammatory drugs (NSAIDs) such as ibuprofen, naproxen sodium, diclofenac, and mefenamic acid. Other allopathic drugs such as combined oral contraceptives, medications that reduce uterine contraction, dietary supplements and narcotics analgesics had found some role in the management of dysmenorrhea [6, 7]. Prevalence of dysmenorrhea varies widely across the world ranging from 15 to 94% [8, 9, 12, 15]. Dysmenorrhea, which can be so debilitating to disrupt the daily activities, work and schooling of post pubescent females is therefore of public health concern. Several studies have reported various risk factors associated with dysmenorrhea which include age less than 20 years, nulliparity, higher socioeconomic status, heavy menses, depression, smoking, anxiety, and lack of physical activity [9, 12, 16]. The socio-economic impact of dysmenorrhea is rather underestimated and not appreciated by the general populace. Previous studies conducted in Ghana on dysmenorrhea were among pre-tertiary students and a limited number of university students all in southern Ghana. This study is therefore aimed at estimating the prevalence of dysmenorrhea, its impact and management among female students of the Tamale campus of the University for Development Studies in Tamale, northern Ghana.

## Method

### Study design and setting

A self-designed semi-structured questionnaire was used in a cross-sectional study involving students of the Tamale campus of the University for Development studies studying to obtained first degrees in Medicine, Nursing, Midwifery, Health Science Education and Community Nutrition. The questionnaire used in the study which occurred between March and April, 2015 was previously piloted among 20 female students across the fields of study involved in the study. Piloting of questionnaire ensured correction of ambiguous and inconsistent questions before it was administered for the actual data collection. A total of 293 out of 389 (75.3%) students returned a well completed questionnaire for this study.

### Study size determination and sampling procedure

Sample size was determined using the Cochran’s (1977) correction formula for categorical data. $$ {n}_1=\frac{n_0}{1+\raisebox{1ex}{${n}_0$}\!\left/ \!\raisebox{-1ex}{$ population$}\right.} $$, n_1_ = required return sample size without estimated response rate factor, n_0_ = required return sample size according to the Cochran formula; $$ {n}_0=\frac{(t)^2\ast (p)\left(1-p\right)}{(d)^2} $$considering sampling error 5% (d = 0.05), the significant level t-value at alpha level of 0.05 (t = 1.96) and 50% of respondents estimated to be experiencing monthly dysmenorrhea (*p* = 0.5). With the study population being 990 from a total female population of 1249 on the Tamale campus of the University for Development Studies and using a possible response rate of 70%, the drawn sample size of 389 was obtained for this study. The number of students to be sampled from each class from the first to the final year except the medical students was based on the population of the class. Only the first to third year pre-clinical level medical students who were on the Tamale campus were involved in the study. From an envelope containing pieces of paper which has names and index numbers of all the female students in each class printed on it, the allotted number of respondents were randomly drawn with replacement by the lead researcher or a volunteer from the class.

### Statistical analysis

Data was analyzed using Microsoft Excel and Graph Pad Prism, Version 5.01 (Graph Pad Software Inc., San Diego CA). Chi square test of independence was used to assess the association between the variables. At a confidence interval of 95%, statistical significance was assumed at *p* < 0.05.

## Results

### Socio-demographic profile

The socio-demographic profile of the respondents is shown in Table 1. In this study, majority, 221 (75.4%) were between ages 20 and 25 years (Mean age = 23 ± 5.07 years; Range = 16 to 48 years), Christians, 208 (71.0%), and spent their vacation in urban areas of Ghana, 181 (61.8%). Again, majority, 161 (54.0%) experienced menarche between ages 13 and 15 (Mean age of menarche = 13.7 ± 1.87 years; Range = 9 to 20 years). At menarche, most respondents, 126 (43.0%) stayed in a self-contained accommodation indicative of their parents and guardians belonging to the middle social class.

**Table 1:** Socio-demographic characteristics of the respondents

Variable	Subgroups	Number of respondents	Percentages
Age (years)	< 20	33	11.3
	20–25	221	75.4
	> 25	39	13.3
Age of menarche	< 13	83	28.3
	13–15	161	54.0
	> 15	49	16.7
Gynecological age (years)	< 5	14	4.8
	5–10	214	73.0
	> 10	65	22.2
Religious affiliation^a^	Christianity	208	71.0
	Islam	79	27.0
Type of accommodation at menarche^a^	Single room	46	15.7
	Chamber and hall	55	18.8
	Several rooms in a compound house	52	17.7
	Self-contained apartment	126	43.0
	Mansion	10	3.4
Area of residence during vacation^a^	Urban area	181	61.8
	Sub-urban	88	30.0
	Rural	21	7.2

^a^There are missing values, so percentage does not add up to 100. The percentages stated are therefore valid percentages

### Characteristics and impact of menstrual pain on respondents

The overall prevalence of dysmenorrhea in this study was 83.6% (*n* = 245). Majority, 138 (56.3%) described their pain as moderate in nature and again for more than half, 143 (58.4%), the pain begins before the outflow of the menstrual blood. The pain lasts less than three days for majority of respondents, 123 (52.6%) which adversely affects the daily activities of more than half, 150 (61.2%) of the respondents. The activity most affected, is attendance to lectures (70.7%). Changes in the breasts such as engorgement, tenderness as well as pain and tingling in the nipples were the most common, 72 (39.1%) associated symptoms of menstruation. Other notable menstruation associated symptoms reported include diarrhea, 29 (15.8%), headache, 34 (18.5%), lethargy, 40 (21.7%), loss of appetite, 35 (19.0%) as well as nausea, 37 (20.1%). Only 40 (16.3%) of persons who suffer menstrual pain had ever reported at a hospital. These characteristics and the impact of menstrual pain on daily activities of respondents are shown in Table 2.

**Table 2:** Characteristics and impact of menstrual pain on respondents

Variable	Subgroup	Number of respondents	Percentage
Presence of dysmenorrhea	Yes	245	83.6
	No	48	16.4
Verbal description of pain	Mild	52	21.2
	Moderate	138	56.3
	Severe	55	22.4
When pain begins	Before blood begins to flow	143	58.4
	During the menstrual flow	98	40.0
	After blood had stopped	0	0.0
Does pain affect daily activities	Yes	150	61.2
	No	95	38.8
How long pain persists (*n* = 234)	< 3 days	123	52.6
	3 to 5 days	100	42.7
	> 5 days	11	4.7
Activities affected by menstrual pain (*n* = 147)	Household chores	82	55.8
	Attendance of lectures	104	70.7
	Concentration at lectures	65	44.2
	Disturbed Sleep	60	40.8
Do you experience other symptoms (*n* = 270)	Yes	184	62.8
	No	86	29.4
Other menstruation associated symptoms experienced	Body weakness	15	8.2
	Diarrhoea	29	15.8
	Breast changes	72	39.1
	Fever	10	5.4
	Headache	34	18.5
	Increased appetite	10	5.4
	Irritable	8	4.3
	Lethargy	40	21.7
	Loss of appetite	35	19.0
	Mood swings	9	4.9
	Nausea	37	20.1
	Pains	29	15.8
	Restlessness	5	2.7
	Vomiting	19	10.3
	Others	22	12.0
Ever taken pain to hospital?	Yes	40	16.3
	No	197	80.4

### Relationship between menstrual characteristics as well as socio-economic factors and occurrence of dysmenorrhea

The relationship between menstrual characteristics as well as socio-economic factors and dysmenorrhea is shown in Table 3. In this study, dysmenorrhea was experienced more by respondents whose age at menarche was less than 13 years than when menarche occurred in later years (89.2% vrs 80.1% - 85.7%; χ^2^ = 3.45, df = 2, *p* = 0.178) Again, dysmenorrhea is more prevalent in Christians than followers of Islam (87% vrs 78.5%, *p* = 0.098); those who live in self-contained apartment at menarche (middle class) than lower or upper class (88.9% vrs 73.9% - 83.6%; χ^2^ = 6.16, df = 4, *p* = 0.188); rural dwellers than urban or semi-urban area dweller (90.5% vrs 82.9% - 85.2%; χ^2^ = 0.927, df = 2, *p* = 0.629); those with moderate menstrual flow than the light or heavy flow respondents (84.5% vrs 62.5% - 84.2%; χ^2^ = 2.77, df = 2, *p* = 0.25); those whose menstrual flow stops after 5 days than those with shorter number of days of flow (88.0% vrs 66.7% - 83.2%, χ^2^ = 2.603, df = 2, *p* = 0.272) and respondents who exercise more often than those who do not exercise (85.6% vrs 82.0%, *p* = 0.053) but these differences were not statistically significant. This study however showed that the chronological age of a female is significantly associated with incidence of dysmenorrhea as persons less than 20 years, significantly experience more menstrual pain than their older colleagues (97.0% vrs 71.8–83.7%; *p* = 0.016).

**Table 3:** Relationship between menstrual characteristics as well as socio-economic factors and dysmenorrhea

Variables	Number of respondents (percentage)	Presence of dysmenorrhea		Chi square (df); *p*-value
		Yes (*n* = 245)	No (*n* = 48)	
Age of respondents (years)	< 20	32 (97.0%)	1 (3.0%)	8.28 (2); 0.016^a^
	20–25	185 (83.7%)	36 (16.3%)	
	> 25	28 (71.8%)	11 (28.2%)	
Age of menarche (years)	< 13	74 (89.2%)	9 (10.8%)	3.45 (2); 0.178
	13–15	129 (80.1%)	32 (19.9%)	
	> 15	42 (85.7%)	7 (14.3%)	
Gynecological age (years)	< 5	12 (85.7)	2 (14.3)	10.09 (2); 0.006^a^
	5–10	187 (87.4)	27 (12.6)	
	> 10	46 (70.8)	19 (29.2)	
Religious affiliation	Christianity	181 (87.0%)	27 (13.0%)	NA (NA); 0.098
	Islam	62 (78.5%)	17 (21.5%)	
Type of accommodation at menarche	Single room	34 (73.9%)	12 (26.1%)	6.16 (4); 0.188
	Chamber and hall	46 (83.6%)	9 (16.4%)	
	Several rooms in a compound house	42 (80.8%)	10 (19.2%)	
	Self-contained apartment	112 (88.9%)	14 (11.1%)	
	Mansion	8 (80.0%)	2 (20.0%)	
Area of residence during vacation	Urban area	150 (82.9%)	31 (17.1%)	0.927 (2); 0.629
	Sub-urban	75 (85.2%)	13 (14.8%)	
	Rural	19 (90.5%)	2 (9.5%)	
Type of menstrual cycle	Regular	178 (84.0%)	34 (16.0%)	NA (NA); 0.572
	Irregular	61 (87.1%)	9 (12.9%)	
Nature of menstrual flow	Light	5 (62.5%)	3 (37.5%)	2.77 (2); 0.250
	Moderate	207 (84.5%)	38 (15.5%)	
	Heavy	32 (84.2%)	6 (15.8%)	
Number of days of flow	< 3 days	6 (66.7%)	3 (33.3%)	2.603 (2); 0.272
	3–5 days	188 (83.2%)	38 (16.8%)	
	> 5 days	44 (88.0%)	6 (22.0%)	
Level of exercising	Exercise often	101 (85.6%)	17 (14.4%)	NA (NA); 0.053
	Does no exercise	131 (82.0%)	41 (18.0%)	

^a^NA – Not applicable since Fisher’s e = exact test was used

### Management of menstrual pain by respondents

As shown in Table 2, up to 83.6% experience dysmenorrhea but just 40 (16.3%) had ever sought treatment from the hospital. How these females manage the pain of menstruation is shown in Table 4. Up to 41.2% of those who experience menstrual pain bear the pain without any effort to reduce or eliminate it. For those who make effort to manage the pain, majority, 105 (72.9%) use one product or procedure while the rest use two or three remedies. Alone or together, taking a bed rest was the most commonly used remedy, 76 (52.8%) while 66 (45.8%) do with allopathic medicines. Majority, 58 (78.9%) of users of allopathic medicine did so without prescription from a hospital. Community pharmacies, 25 (43.1%) and Over-the-counter medicine sellers shop, 24 (41.4%), were the most common outlets for the procurement of these self-prescribed orthodox medications. Up to 50 (86.2%) of users of self- prescribed medications agree or strongly agree that they always obtain relieve from the menstrual pains on using these medications.

**Table 4:** Management of menstrual pain by respondents

Variable	Subgroup	Number of respondents	Percentage
Ever taken pain to hospital?	Yes	40	16.3
	No	197	80.4
How did you manage your pain in the last three months (*n* = 144 for those who attempted to manage the pain)	Did nothing (n = 245)	101	41.2
	Consulted a physician	11	7.6
	Took a bed rest	76	52.8
	Took orthodox medication	66	45.8
	Took herbal preparation	5	3.5
	Used a heat pad	12	8.3
	Exercised	21	14.6
If you took medication, were they prescribed? (*n* = 66)	Yes	8	12.1
	No	58	78.9
Source of self-medicated drugs	Community pharmacy	25	43.1
	Over-the-counter medicine sellers’ shop	24	41.4
	Friends and relatives	6	10.3
	Others	2	3.4
Always got relieved after self-medicating.	Strongly agree	18	31.0
	Agree	32	55.2
	Uncertain	3	8.6
	Disagree	5	5.2

### Relationship between bio-data, menstrual characteristics, attitudes and severity of the dysmenorrhea

Respondents between the ages 20 and 25 experienced increasing intensity of pain (Mild = 69.2%, Moderate = 75.4% and Severe = 81.8%) but the difference in relation to other age brackets was not significant (χ^2^ = 3.104, df = 4, *p* = 0.540). Menstrual duration (χ2 = 7.222, df = 4, *p* = 0.125), and nature of menstrual flow (χ^2^ = 9.005, df = 6, *p* = 0.173) showed no association with intensity of pain experienced by respondents. There was however, a significant association between the following variable and likelihood of experiencing the severest form of dysmenorrhea; irregular menstrual flow (Mild = 9.6%, Moderate = 27.3% and Severe = 36.4%; χ^2^ = 10.54, df = 2, *p* = 0.005), tendency to seek treatment at the hospital (Mild = 6.0%, Moderate = 6.1% and Severe = 52.7%; χ^2^ = 65.61, df = 2, *p* < 0.0001) and self-medication (Mild = 10.0%, Moderate = 22.1% and Severe = 56.4%; χ^2^ = 32.77, df = 2, p, 0.0001). Table 5 showed the relationship between bio-data, menstrual characteristics, attitudes and severity of the dysmenorrhea of the respondents.

**Table 5:** Relationship between bio-data, menstrual characteristics, attitudes and severity of the dysmenorrhea

Variable	Subgroup	Severity of dysmenorrhea			χ^2^ (df)	*p*-value
		Mild	Moderate	Severe		
Age of respondents	< 20	7 (13.5)	19 (13.8)	6 (10.9)	3.104 (4)	0.540
	20–25	36 (69.2)	104 (75.4)	45 (81.8)		
	> 25	9 (17.3)	15 (10.9)	4 (7.3)		
Age of menarche	< 13	13 (25.0)	43 (31.2)	18 (32.7)	3.258 (4)	0.516
	13–15	26 (50.0)	75 (54.3)	28 (50.9)		
	> 15	13 (25.0)	20 (14.5)	9 (16.4)		
Gynecological age (years)	< 5	4 (7.7)	5 (3.6)	3 (5.5)	1.996 (4)	0.737
	5–10	37 (71.2)	109 (79.0)	41 (74.5)		
	> 10	11 (21.2)	24 (17.4)	11 (20.0)		
Menses duration	< 3 days	1 (2.0)	3 (2.2)	2 (3.8)	7.222 (4)	0.125
	3–5 days	41 (80.4)	112 (83.6)	35 (66.0)		
	> 5 days	9 (17.6)	19 (14.2)	16 (30.2)		
Menstrual pattern	Regular	47 (90.4)	96 (72.7)	35 (63.6)	10.54 (2)	0.005^a^
	Irregular	5 (9.6)	36 (27.3)	20 (36.4)		
Level of menstrual flow	Light	2 (3.8)	2 (1.5)	1 (1.8)	9.005 (6)	0.173
	Moderate	47 (90.4)	119 (86.9)	41 (74.5)		
	Heavy	3 (5.8)	16 (11.7)	13 (23.6)		
Visited hospital due to the pain	Yes	3 (6.0)	8 (6.1)	29 (52.7)	65.61 (2)	< 0.0001^a^
	No	47 (94.0)	124 (93.3)	26 (47.3)		
Practised self- medication?	Yes	5 (10.0)	30 (22.1)	31 (56.4)	32.77 (2)	< 0.0001^a^
	No	45 (90.0)	106 (77.9)	24 (43.6)		

^a^statistically significant

### Medications used in the management of menstrual pain

Different types of medications were used by the respondents for the relief of the menstrual pains as shown in Table 6. Alone or present in a compound preparation, paracetamol was the most commonly, 43 (41.3%) used medication*.* Non-steroidal anti-inflammatory drugs; diclofenac (18.3%), ibuprofen (10.6%), mefenamic acid preparations such as ladinax, menstropain, ponstan, laxinas (10.3%) were also used by some of the respondents. Antispasmodic, hyoscine butylbromide was used by a few of the respondents (6.7%). In most times, 47 (50.5%) that a respondent engaged in self -medication, they depended on their own knowledge but in 13 (14.0%) cases of self -medication, they got the medication based on the recommendation of the pharmacist. Comparing the doses stated by the respondents with that in the British National Formulary, the dosage regimen applied by the respondents was incorrect in most cases (57.7%).

**Table 6:** Drugs used in the management of the pain in the last three menstrual cycle

Variable	Subgroup	Number	Percentage
Medication used for self –medication	Paracetamol	43	41.3
	Mefenamic acid preparation	11	10.6
	Diclofenac	19	18.3
	Hyoscine butylbromide (Buscopan)	7	6.7
	Ibuprofen	11	10.6
	Herbal preparations	4	3.8
	Others	9	8.7
Persons who recommended these drugs	Self	47	50.5
	Prescriber	9	9.7
	Nurse	7	7.5
	Mother	8	8.6
	Friends	9	9.7
	Pharmacist	13	14.0
Dosage assessment	Correct	44	42.3
	Incorrect	60	57.7

### Classes of medication for managing dysmenorrhea based on severity of pain

**Fig. 1:**
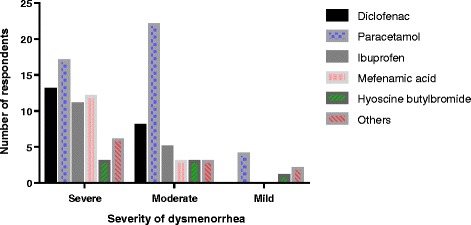
Classes of medication for managing dysmenorrhea based on severity of pain

The medications used for managing dysmenorrhea based on severity of pain are presented in Fig. 1. The number of occasions that the various classes of medication were used by the respondents based on whether the pain was mild, moderate or severe were diclofenac (0, 8, 13), paracetamol (4, 22, 17), ibuprofen (0, 5, 11), mefenamic acid (0, 3, 12), hyoscine butylbromide (1, 3, 3) and others (2, 3, 6). Paracetamol preparations were the most commonly used (38.1%) class of medication for all the types of dysmenorrhea and followed by diclofenac preparations (18.6%).

## Discussions

Menstruation is considered a private issue in many cultures hence its associated complaints are borne silently by most post-pubescent females. For some women, they experience a monthly ritual of lower abdominal pain during menstruation known as dysmenorrhea. This study found a high prevalence of dysmenorrhea (83.6%) which is comparable to 85.0% recorded in United States of America (USA) [17] and 84.1% in Italy [9]. An earlier study among secondary school students in Accra, Ghana recorded a lower prevalence rate of 74.4% [18]. Studies in some other countries recorded lower rates between 38.1 and 76.0% [1, 6, 12, 19, 20] but higher prevalence rates of 92.5 and 94% were respectively reported in Taiwan and Oman [8, 21]. The varying prevalence rates of dysmenorrhea across the world could be attributed to the use of different categories of subjects as well as the lack of a universally accepted definition of dysmenorrhea. Majority of respondents in this study described their menstrual pain as moderate similar to studies in Italy and Oman [8, 9] but in some studies, majority classified their pain as mild [6, 11]. Pain perception and expression of pain is influenced by genetics, psychological, developmental, familial, social and cultural factors [22–24]. Therefore, the aforementioned factors as well as variability of pain threshold of the different categories of respondents who participated in all these studies could account for the differences in the description of their pains. Dysmenorrhea in this study affected the daily life activities of two-thirds of the female students including school attendance as reported similarly in several studies across the world [1, 6, 8, 9, 12, 13]. Attending lectures was the most disrupted daily life activity for respondents in this study and even if they make it to the lectures, their concentration was greatly disturbed due to the dysmenorrhea. Just as reported in several studies on dysmenorrhea, two-third of subjects in this study experience menstruation associated symptoms such as diarrhea, headaches, lethargy, loss of appetite, nausea, joint and body pains, vomiting but the most common symptom was changes in the breast such as tenderness, heaviness, engorgement, as well as tingling and painful nipples which occurred in 39.1% of respondents [1, 12, 21]. This study did not find any significant association between incidence of dysmenorrhea and socio-demographic characteristics such religious affiliation, socio-economic status at menarche and locality of residence. This study however found a significant association between a respondent experiencing dysmenorrhea and the chronological as well as gynecological ages which was also reported in other studies which showed that incidence of dysmenorrhea decreases with increasing chronological age or gynecological age [16, 25]. Type of menstrual cycle, nature of the menstrual flow, number of days of menstrual flow and exercise did not predict the presence or absence of dysmenorrhea in this study. It would have been expected that the undesirable effects of dysmenorrhea and menstruation associated symptoms on the activities of respondents, would cause them to be eager to visit the hospital but only 16.3% ever did so. Similar hospital attendance rates between 12.1 and 18.0% were reported in Iran, Malaysia, Nigeria, Turkey and the USA [2, 10, 14, 17, 26]. A mere 3% was even recorded in Oman [8]. Self-treatment of menstrual pain using mostly non-steroidal anti-inflammatory drugs and antispasmodic drugs seem to be the most common practice in many countries [2, 6, 8, 10, 12, 14, 26, 27]. The self-treatment of dysmenorrhea by many women rather than visit a hospital is because many consider the pain as normal thus not worth taking to a hospital where they may join long queues to consult a physician. Again, many who self-medicate get relieve from the use of these medications as shown in this study. In this study, up to four-fifth of users of non-prescribed medications stated that they always get relieved of their pains after using these medications although the dosage regimen followed by three-fifth of the users were not appropriate. The suggestion by 86.2% of the females that they get relieved even with the high level of incorrect dosage regimen could be due to a challenge of recall of the correct dosage regimen or the healing could be due to the placebo effect [28]. Contrary to other studies, this study did not find any significant relationship between pain intensities suffered by respondents and their biological age, age at menarche, gynecological age, nature and duration of menstrual flow [1, 6, 25, 29]. There was rather an association between the menstrual pattern and severity of dysmenorrhea as recorded in other studies in which persons with irregular menstrual pattern suffered a more severe form of menstrual pain (χ^2^ = 10.54; df = 2; *p* = 0.005) [6]. This study also found that females with severe dysmenorrhea exhibited a significantly greater tendency to seek treatment at the hospital (χ^2^ = 65.61; df = 2; *p* < 0.0001) or self-medicate (χ^2^ = 32.77; df = 2; p < 0.0001). The classes of drugs used for the dysmenorrhea did not significantly differ. Paracetamol (Acetaminophen) or its combination preparation was the most patronized analgesic notwithstanding the severity of the menstrual pain. The use of paracetamol for the management of dysmenorrhea was also reported in some earlier studies [8, 10, 26, 27]. Paracetamol, although exhibits a weaker analgesic effect than NSAIDs, it is better tolerated and has better safety profile [30] and could be appropriate for managing dysmenorrhea in females who are at risk of peptic ulcer or asthma; conditions for which NSAIDs are contraindicated.

## Conclusions

Dysmenorrhea is a major menstruation related complaint among the female university students in northern Ghana. A significant association exists between the chronological and gynecological ages of respondents with younger students experiencing dysmenorrhea a lot more. Irregular menstruation is significantly associated with the severest form of dysmenorrhea but the chronological age, age at menarche or socio-economic disposition of the respondent do not. Dysmenorrhea and the menstruation associated symptoms adversely affect the daily lives of the females with some missing school. Although bed rest was the most applied modality, allopathic medication especially paracetamol preparations were used especially by those with moderate form of dysmenorrhea.

## Abbreviations

NSAIDs: Non-steroidal anti-inflammatory drugs
USA: United States of America
